# Measurement properties of patient-reported outcome measures (PROMs) in patients with hyperhidrosis: protocol for a systematic review

**DOI:** 10.1186/s13643-021-01701-w

**Published:** 2021-06-02

**Authors:** Michaela Gabes, Helge Knüttel, Christian J. Apfelbacher

**Affiliations:** 1 Institute of Social Medicine and Health Economics, Otto-von-Guericke-University Magdeburg, Magdeburg, Germany; 2 Medical Sociology, Department of Epidemiology and Preventive Medicine, University of Regensburg, Regensburg, Germany; 3 University Library, University of Regensburg, Regensburg, Germany

**Keywords:** Hyperhidrosis, Patient-reported outcome measures, COSMIN, Measurement properties, Validity, Reliability, Responsiveness

## Abstract

**Background:**

Hyperhidrosis is a chronic skin condition that impairs the patient’s quality of life (QoL). There are several patient-reported outcome measures (PROMs) for patients affected by hyperhidrosis available; however an evidence-based assessment of their quality has not been undertaken so far.

**Objective:**

In our systematic review, we aim to identify all existing PROMs that were developed and/or validated for measuring patient-reported outcomes in patients with hyperhidrosis and assess their measurement properties in a transparent and structured way to give a recommendation for future clinical research.

**Methods/design:**

Our systematic review aims to contain all PROMs developed and/or validated for patients with hyperhidrosis. We will perform a highly sensitive, systematic literature search including the databases MEDLINE (Ovid), EMBASE (Ovid), and Science Citation Index Expanded and Social Sciences Citation Index (Web of Science). Especially studies which evaluate, describe, or compare measurement properties of PROMs for patients with hyperhidrosis will be considered as eligible. Two independent reviewers will judge the eligibility of the studies found in the literature search. The study and PROM characteristics will be summarized in evidence tables. The methodological quality of each study will be assessed using the COnsensus-based Standards for the selection of health Measurement INstruments (COSMIN) Risk of Bias checklist. We will apply predefined and consensus-based quality criteria for good measurement properties. Subsequently, the quality of the evidence will be graded. Furthermore, aspects on interpretability and feasibility will be described. A final recommendation will be given.

**Discussion:**

In our systematic review, we aim to provide a comprehensive description of the quality of all existing PROMs for patients with hyperhidrosis. The assessment of measurement properties, interpretability, and feasibility will serve as a guidance regarding the selection of PROMs for future clinical hyperhidrosis trials.

**Systematic review registration:**

PROSPERO CRD42020170247

**Supplementary Information:**

The online version contains supplementary material available at 10.1186/s13643-021-01701-w.

## Background

Hyperhidrosis is a term used to describe a skin condition which is characterized by excessive sweating beyond what is physiologically necessary [1]. It can be either classified as primary or secondary hyperhidrosis, based on the cause of sweating [2]. In the USA, a prevalence of less than 5% is reported [1, 3, 4]; however, this disease is often underdiagnosed and therefore underreported [2]. Quality of life (QoL) is negatively affected in patients with hyperhidrosis [2, 5].

To foster involvement of patients in both clinical research as well as routine health care, the use of patient-reported outcome measures (PROMs) has steadily increased in the past decades. These instruments reflect the patient’s perspective of how they perceive their health status and whether health care interventions have been effective. PROMs are self-completed questionnaires measuring, e.g., health-related QoL or health status [6]. In hyperhidrosis, several QoL instruments, from hyperhidrosis-specific over skin-specific to more generic tools, are used to evaluate the condition [7].

In a recent narrative review [8], 22 QoL tools in the context of hyperhidrosis were identified via a comprehensive literature search. Four of these instruments, the Hyperhidrosis Disease Severity Scale (HDSS), the Dermatology Life Quality Index (DLQI), the Hyperhidrosis Quality-of-Life Questionnaire (HQLQ), and the Hyperhidrosis Quality of Life Index (HidroQoL) were commented on a second step by a group of patient advisors (n = 4) and one dermatologist. This group preferred the HidroQoL [9] over the other instruments and suggested that this instrument should be the primary outcome in future intervention studies.

However, it is important in clinical research to select measurement instruments which are reliable, valid, responsive, and feasible. The selection of instruments should be based on complete information regarding these measurement properties and the quality of the underlying research. The international COSMIN (COnsensus-based Standards for the selection of health Measurement INstruments) initiative (https://www.cosmin.nl/) aims to improve the selection of the most suitable PROM and therefore, developed a methodology and practical tools for the assessment of the quality of single PROMs and their development and validation studies. This recent narrative review based their recommendation only on the judgement of a small group [8]. A systematic comparison of the existing PROMs for patients with hyperhidrosis and a judgement of the quality of these PROMs using the COSMIN methodology to give an evidence-based recommendation have not been undertaken yet.

## Aim and objectives

Our overall aim is to critically appraise, compare, and summarize the quality of all existing PROMs in patients with hyperhidrosis.

More specifically, our objectives are the following:

1. To systematically assess the measurement properties of PROMs in hyperhidrosis
2. To identify PROMs in hyperhidrosis

- That meet the predefined criteria to be recommended in future hyperhidrosis trials
- That have the potential to be recommended in the future depending on the results of further validation studies
- That do not meet the predefined criteria to be recommended and therefore should not be used anymore

Furthermore, we will explore all existing PROMs for underlying constructs, e.g., physical health, social health, and mental health. Dodd et al. [10] explored health research vocabularies and developed a pilot classification system of outcomes to prevent inconsistency and variation in how outcomes are described. We will classify all constructs according to Dodd et al. and try to recommend one best PROM per construct.

## Methods/design

### Protocol and registration

The present systematic review will be conducted in accordance with the recommendations from the Preferred Reporting Items for Systematic Reviews and Meta-Analyses Protocols (PRISMA-P) statement [11] and the COSMIN guideline and manual for systematic reviews of PROMs [12, 13]. The current PRISMA-P checklist is available as an Additional file 1 to this protocol. This protocol has been submitted to the International Prospective Register of Systematic Reviews (PROSPERO): CRD42020170247.

### Literature search

A systematic literature search will be performed in the bibliographic databases MEDLINE (via Ovid, 1946–present), EMBASE (via Ovid, 1974–present), Science Citation Index Expanded (Web of Science, 1965–present), and Social Sciences Citation Index (Web of Science, 1990–present). The search strategy will be comprised of the following search elements [12]:

A. Target population: patients with hyperhidrosis. In order to reach maximal sensitivity a broad compilation of controlled vocabulary and free text terms will be used.
B. Construct of interest: all patient-reported outcome measures regardless of the underlying construct. For optimal sensitivity the search strategy of this search element will be based on a combination of the PubMed filter “Quality of life (QoL)” of Vissers and de Vries [14], the PubMed filter “Patient reported outcome measures (PROMs)” of Jansma and de Vries [15], and additional search terms from the “PROM group construct and instrument type filter” of Mackintosh et al. [16]. Patient-reported outcome measures is a broad term and it includes measures of QoL or health status [6, 17].
C. Measurement properties: the validated and sensitive search filter (recommended by the COSMIN group [13]) for finding studies on measurement properties developed by Terwee et al. [18] will be used. We will employ the translation of the original PubMed filter to Ovid MEDLINE by Alberta University [19].
D. Feasibility of PROMs: the search strategy for this element will be based on the search terms for the concept ‘feasibility’ of Heinl et al. [20] (included in their search statement #1, Additional file 2).
E. Individual PROMs: a list of known relevant PROMs in the context of hyperhidrosis [8]. This list will be comprised of two parts, a list of hyperhidrosis-specific PROMs (E1) and a list of general purpose PROMs (E2). From the result set E2, only the intersect with set A (target population) will be used.
F. Exclusion filter: this will be the exclusion filter from Terwee et al. [18] for a number of irrelevant publication types and for animal-only studies.
G. Language filter: only reports in English, German, French, or Italian will be included.

**Fig. 1:**
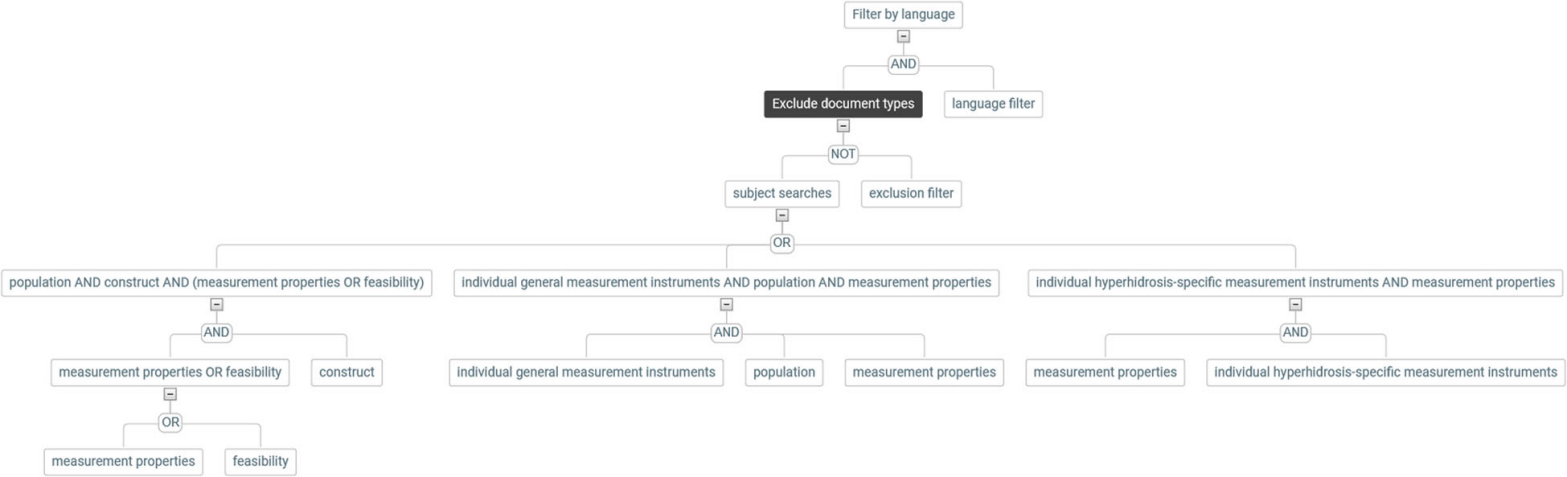
Tree view of the structure of the database search strategy

The search elements will be combined as follows in order to identify all articles on the measurement properties or the feasibility of PROMs in the context of hyperhidrosis. From these records, the exclusion filter will remove irrelevant publication types as well as animal-only studies: (((A AND B AND (C OR D)) OR (C AND E)) NOT F) AND G, or in words: (((population AND construct AND (measurement properties OR feasibility)) OR (individual PROMs AND measurement properties)) NOT (exclusion filter)) AND language filter. A graphical overview of the search structure in form of a tree is given in Fig. 1.

In addition, databases specific for PROMs will be searched for records relevant to the target population: PROQOLID (https://eprovide.mapi-trust.org/about/about-proqolid), the COSMIN database of systematic reviews of outcome measurement instruments (http://www.cosmin.nl/database-of-systematic-reviews.html), the Test Archive of Leibniz Institute for Psychology Information (https://www.testarchiv.eu/) and the PubPsych search engine (https://pubpsych.zpid.de/pubpsych/). In addition to the electronic search, hand searching of the reference lists of the studies included and key articles on this topic will be searched.

The search strategy for MEDLINE can be found as an Additional file 2 to this protocol. The search strategy will be translated to the other databases choosing appropriate syntax and index terms.

Subsequently, the bibliographic databases and the databases specifically on PROMs will be searched again with the names of hyperhidrosis-specific PROMs found during the initial search.

There will be no restrictions regarding publication date. Only papers in English, German, French or Italian will be included. All records from searching will be exported to EndNote X9 for further processing including deduplication and title/abstract screening. In a second step, full texts will be reviewed and data extracted.

### Eligible studies

The eligibility criteria are in agreement with the COSMIN guideline for systematic reviews of patient-reported outcome measures [13]. The included studies should concern PROMs. The evaluation of measurement properties, the development of a PROM, or the evaluation of the interpretability of the PROMs of interest should be the principal aim of selected studies. Studies that only use the PROM to measure the outcome or in which the PROM is used for the validation of another instrument will be excluded. Only full-text articles will be included because abstracts provide quite often very limited information on the design of a study. Studies that concern the development (“development paper”) and/or the evaluation of the measurement properties (“validation paper”) of PROMs are included as well. At least 50% of the study sample must consist of hyperhidrosis patients to fulfill the eligibility criteria (Table 1).

**Table 1:** Inclusion and exclusion criteria

	Inclusion criteria	Exclusion criteria
Population	Patients with hyperhidrosis (any severity)	Populations with other skin diseases
Study design	PROM development study, validation study	All other study designs
Outcome	All patient-reported outcomes	Non-patient-reported outcomes, such as biomarkers or physiology of the skin
Type of measurement instrument	Patient-reported measurement instruments	All others
Publication type	Articles with available full text	Abstracts

### Study selection

Titles and abstracts found in the literature search will be independently judged by two reviewers. For the remaining titles and abstracts, full-text articles will be searched and judged for eligibility also by two reviewers independently. If any disagreement occurs, consensus will be reached by consulting a third reviewer. If at least one reviewer considers a study as relevant based on the abstract, or in case of doubt, the full-text article needs to be screened.

### Data extraction

The extracted relevant data will be summarized in evidence tables, one evidence table with the characteristics of the included PROMs, and one table with the characteristics of the included study populations. If general characteristics of a PROM cannot be extracted, the original development paper will be consulted [12].

### Assessment of measurement properties, further characteristics of PROMs, and quality of the PROMs

The measurement properties of the single PROMs will be evaluated in the following order:

- Evaluation of the content validity
- Evaluation of internal structure including structural validity, internal consistency, and cross-cultural validity/measurement invariance
- Evaluation of remaining measurement properties including reliability, measurement error, criterion validity, hypotheses testing for construct validity, and responsiveness

All measurement properties will be evaluated following three sub steps [12], except for the measurement property “criterion validity” since there exists no gold standard for QoL. For construct validity and responsiveness, we will formulate hypotheses to evaluate the results against.

First, we will evaluate the methodological quality of the included studies using the COnsensus-based Standards for the selection of health Measurement INstruments (COSMIN) Risk of Bias checklist consisting of ten boxes: PROM development, content validity, structural validity, internal consistency, cross-cultural validity/measurement invariance, reliability, measurement error, criterion validity, hypotheses testing for construct validity, and responsiveness [21]. Only those boxes for the measurement properties that are assessed in one article will be completed. The COSMIN taxonomy will be used to decide which measurement property has been evaluated. The standards include both preferred statistical methods based on Classical Test Theory (CTT) and Item Response Theory (IRT) or Rasch analyses.

Definitions of all included measurement properties are included in the paper of Mokkink et al. [22]. Content validity can be seen as the most important measurement property, because the items of a PROM have to be relevant, comprehensive, and comprehensible regarding the population and construct of interest. If there is high-quality evidence for insufficient content validity, the PROM will not be further assessed and directly categorized as C, i.e., the PROM should not be recommended for use. Each study will be rated on a 4-point rating scale (that is, “inadequate,” “doubtful,” “adequate,” and “very good”). The overall quality of a study is determined by the lowest rating of any standard in the box, i.e., “the worst score counts” principle [21]. Each study on a measurement property will be assessed separately and all measurement properties of each study will be rated as either very good, adequate, doubtful or inadequate [12].

Second, we will apply criteria for good measurement properties (quality criteria). The updated criteria for good measurement properties recommended by the COSMIN group [13] are presented in Table 2. The result of each single study will be rated as either sufficient (+), insufficient (−), or indeterminate (?) [12].

**Table 2:** Updated criteria for good measurement properties [13], based on, e.g., Terwee et al. [23] and Prinsen et al. [24]

Measurement property	Rating	Criteria
Structural validity	+	**CTT** CFA: CFI or comparable measure > 0.95 OR RMSEA < 0.06 OR SRMR < 0.08^a^ **IRT/Rasch** No violation of unidimensionality^b^: CFI or TLI or comparable measure > 0.95 OR RMSEA < 0.06 OR SMRM < 0.08 *AND* No violation of local independence: residual correlations among the items after controlling for the dominant factor < 0.20 OR Q3’s < 0.37 *AND* No violation of monotonicity: adequate looking graphs OR item scalability > 0.30 *AND* Adequate model fit IRT: *χ*^2^ > 0.001 Rasch: infit and outfit mean squares ≥ 0.5 and ≤ 1.5 OR *Z*-standardized values > − 2 and < 2
	?	CTT: not all information for ‘+’ reported IRT/Rasch: model fit not reported
	−	Criteria for ‘+’ not met
Internal consistency	+	At least low evidence^c^ for sufficient structural validity^d^” AND Cronbach’s alpha(s) ≥ 0.70 for each unidimensional scale or subscale^e^
	?	Criteria for “At least low evidence^c^ for sufficient structural validity^d^” not met
	−	At least low evidence^c^ for sufficient structural validity^d^ and Cronbach’s alpha(s) < 0.70 for each unidimensional scale or subscale^e^
Reliability	+	ICC or weighted Kappa ≥ 0.70
	?	ICC or weighted Kappa not reported
	−	ICC or weighted Kappa < 0.70
Measurement error	+	SDC or LoA < MIC^d^
	?	MIC not defined
	−	SDC or LoA > MIC
Hypotheses testing for construct validity	+	The result is in accordance with the hypothesis^f^
	?	No hypothesis defined (by the review team)
	−	The result is not in accordance with the hypothesis^f^
Cross-cultural validity/measurement invariance	+	No important differences found between group factors (such as age, gender, language) in multiple group factor analysis OR no important DIF for group factors (McFadden’s *R*^2^ < 0.02)
	?	No multiple group factor analysis OR DIF analysis performed
	−	Important differences between group factors OR DIF was found
Criterion validity	+	Correlation with gold standard ≥ 0.70 OR AUC ≥ 0.70
	?	Not all information for ‘+’ reported
	−	Correlation with gold standard < 0.70 OR AUC < 0.70
Responsiveness	+	The result is in accordance with the hypothesis^f^ OR AUC ≥ 0.70
	?	No hypothesis defined (by the review team)
	−	The result is not in accordance with the hypothesis^f^ OR AUC < 0.70

“+” = sufficient, “−” = insufficient, “?” = indeterminate

*AUC* area under the curve, *CFA* confirmatory factor analysis, *CFI* comparative fit index, *CTT* classical test theory, *DIF* differential item functioning, *ICC* intraclass correlation coefficient, *IRT* item response theory, *LoA* limits of agreement, *MIC* minimal important change, *RMSEA* root mean square error of approximation, *SEM* standard error of measurement, *SDC* smallest detectable change, *SRMR* standardized root mean residuals, *TLI* Tucker-Lewis index

^a^To rate the quality of the summary score, the factor structure should be equal across studies; ^b^Unidimensionality refers to a factor analysis per subscale, while structural validity refers to a factor analysis of a (multidimensional) patient-reported outcome measure

^c^As defined by grading the evidence according to the GRADE approach

^d^This evidence may come from different studies

^e^The criteria ‘Cronbach’s alpha < 0.95’ was deleted, as this is relevant in the development phase of a PROM and not when evaluating an existing PROM

^f^The results of all studies should be taken together and it should then be decided if 75% of the results are in accordance with the hypotheses

Third, we aim to summarize the evidence per measurement property per PROM, rate the overall result against criteria for good measurement properties, and grade the quality of the evidence by using the GRADE approach. Here, we focus on the PROM and not as in the previous steps on the single studies [12].

The third sub step includes several further substeps.

First, we will have to decide if the results of all studies per measurement property are consistent or not [12].

If they are consistent, they can be pooled or summarized and an overall rating as either sufficient (+), insufficient (−), or indeterminate (?) can be provided after the comparison against the quality criteria. Finally, their quality of the evidence will be graded [12].

If the results are inconsistent, we will look for explanations for inconsistency.

- If an explanation is found, the different results will be summarized (e.g., per subgroup of consistent results) followed by an overall rating for the specific measurement property. It should be considered that high-quality studies provide more evidence than low quality studies when determining the overall rating [12].
- If no explanation for inconsistency is found, the overall rating could be either inconsistent (±) or based on the majority of the results and therefore downgraded for inconsistency (see GRADE approach explained below) [12].

Second, we will pool the results quantitatively or summarize them qualitatively in Summary of Findings (SoF) Tables. Each measurement property per PROM in one table [12].

Third, each pooled or summarized result will be again rated against the quality criteria (Table 2) to obtain an overall rating for the pooled or summarized result as either sufficient (+), insufficient (−), inconsistent (±), or indeterminate (?). This rating will be added to the Summary of Findings Tables [12].

Fourth, the quality of the evidence will be graded to define whether the pooled or summarized result is trustworthy. It is important to consider the quality of evidence because insufficient attention to quality of evidence can lead to inappropriate recommendations that may have negative impacts for the patients. The recognition of the quality of evidence can help to prevent misguided recommendations [25]. Using the GRADE approach, we will determine whether confidence in estimates of true measurement properties is given. For this systematic review, we use a GRADE approach with four GRADE factors: risk of bias, inconsistency, imprecision, and indirectness. Those depend on four levels of quality evidence (i.e., high, moderate, low, or very low) which are specified by the GRADE approach [13]. If the results do not seem trustworthy, the quality of evidence will be downgraded. Each PROM will be graded separately. If the overall rating for a measurement property is indeterminate (?), the quality of the PROMs cannot be judged and therefore the quality of evidence will not be graded [13]. All results will be added to the Summary of Findings Tables as well [12].

Interpretability and feasibility which are also important for a recommendation will be described after the evaluation of the measurement properties. Interpretability means the degree to which qualitative meaning can be assigned to a PROM’s quantitative score. Feasibility contains aspects of the ease of application (e.g., costs, length, ease of administration) [12].

### Generating recommendations for the use of PROMs in hyperhidrosis

The included PROMs will be classified into three categories [12]:

A. PROMs with evidence for sufficient content validity (any level) AND at least low-quality evidence for sufficient internal consistency: recommended for use
B. PROMs categorized not in A or C: further validation needed
C. PROMs with high-quality evidence for an insufficient measurement property: should not be recommended for use

If only PROMs of category B will be found, the PROM with the best evidence for content validity can be preliminarily recommended for use, until further evidence is given [12].

## Discussion

A broad and accurate assessment of the measurement properties of existing PROMs for patients with hyperhidrosis has not been done so far and is planned for this systematic review. Our aim is to recommend the best PROM(s) for each of the constructs identified in the literature search. We will fully report each step of the systematic review in a transparent and structured way. Furthermore, we will involve at least two independent reviewers to assure quality and reduce variability in the assessments. The COSMIN Risk of Bias checklist is a recently developed and well-established method to assess the methodological quality of the studies. By using the GRADE approach, we ensure the certainty of the evidence. If concerns about the quality of the evidence are given (e.g., low sample size or unexplained inconsistency), the quality of evidence will be downgraded to prevent errors in recommendation [12]. Interpretability and feasibility of the PROMs will be considered as well since it is essential for a recommendation to have information on these two aspects. Interpretability gives information on how single scores or change scores can be interpreted, i.e., the degree to which it is clear what the scores mean [26]. We will look for information on feasibility during the review process and we will use them for our final recommendation. Feasibility aspects can be decisive for a recommendation when there are two or more PROMs that are difficult to differentiate in terms of quality. It is unclear if we are able to arrive at recommending several good PROMs or not. It could also happen that we can only provide guidance for future validation studies. A potential limitation of this study may be the fact that we are only considering articles in specific languages (English, German, French, and Italian). Unfortunately, we cannot consider articles in Spanish which is also one of the most spoken languages in the world. This can cause an interesting article to be lost.

## Supplementary Information

**Additional file 1:.** PRISMA-P 2015 Checklist.

**Additional file 2:.** MEDLINE search strategy.

## Data Availability

Not applicable

## Abbreviations

COSMIN: Consensus-based Standards for the selection of health Measurement INstruments
PRISMA-P: Preferred Reporting Items for Systematic Reviews and Meta-Analyses Protocols
PROM: Patient-reported outcome measure
PROSPERO: International Prospective Register of Systematic Reviews
QoL: Quality of Life
